# Large-scale production of myco-fabricated ZnO/MnO nanocomposite using endophytic *Colonstachys rosea* with its antimicrobial efficacy against human pathogens

**DOI:** 10.1038/s41598-024-51398-9

**Published:** 2024-01-10

**Authors:** Shahira H. EL-Moslamy, Ahmed Ibrahim Abd-Elhamid, Gomaa El Fawal

**Affiliations:** 1 Department of Bioprocess Development (BID), Genetic Engineering and Biotechnology Research Institute (GEBRI), City of Scientific Research and Technological Applications (SRTA-City), New Borg El Arab City, Alexandria, 21934 Egypt; 2 Composites and Nanostructured Materials Research Department, Advanced Technology and New Materials Research Institute (ATNMRI), City of Scientific Research and Technological Applications (SRTA-City), New Borg El Arab, Alexandria, 21934 Egypt; 3 Polymer Materials Research Department, Advanced Technology and New Materials Research Institute (ATNMRI), City of Scientific Research and Technological Applications (SRTA-City), New Borg El Arab, Alexandria, 21934 Egypt

**Keywords:** Biological techniques, Biophysics, Biotechnology, Nanoscale materials

## Abstract

In this study, a ZnO/MnO nanocomposite was myco-fabricated using the isolated endophytic *Clonostachys rosea* strain EG99 as the nano-factory. The extract of strain EG99, a reducing/capping agent, was successfully titrated with equal quantities of Zn(NO_3_)_2_·6H_2_O and Mn(NO_3_)_2_·6H_2_O (precursors) in a single step to fabricate the rod-shaped ZnO/MnO nanocomposite of size 6.22 nm. The ZnO/MnO nanocomposite was myco-fabricated in 20 min, and the results were validated at 350 and 400 nm using UV–Vis spectroscopy. In a 7-L bioreactor, an industrial biotechnological approach was used to scale up the biomass of this strain, EG99, and the yield of the myco-fabricated ZnO/MnO nanocomposite. A controlled fed-batch fermentation system with a specific nitrogen/carbon ratio and an identical feeding schedule was used in this production process. Higher yields were obtained by adopting a controlled fed-batch fermentation approach in a 7-L bioreactor with a regular feeding schedule using a nitrogen/carbon ratio of 1:200. Overall, the fed-batch produced 89.2 g/l of biomass at its maximum, 2.44 times more than the batch's 36.51 g/l output. Furthermore, the fed-batch's maximum ZnO/MnO nanocomposite yield was 79.81 g/l, a noteworthy 14.5-fold increase over the batch's yield of 5.52 g/l. Finally, we designed an innovative approach to manage the growth of the endophytic strain EG99 using a controlled fed-batch fermentation mode, supporting the rapid, cheap and eco-friendly myco-fabrication of ZnO/MnO nanocomposite. At a dose of 210 µg/ml, the tested myco-fabricated ZnO/MnO nanocomposite exhibited the maximum antibacterial activity against *Staphylococcus aureus* (98.31 ± 0.8%), *Escherichia coli* (96.70 ± 3.29%), and *Candida albicans* (95.72 ± 0.95%). At the same dose, *Staphylococcus aureus* biofilm was eradicated in 48 h; however, *Escherichia coli* and *Candida albicans* biofilms needed 72 and 96 h, respectively. Our myco-fabricated ZnO/MnO nanocomposite showed strong and highly selective antagonistic effects against a variety of multidrug-resistant human pathogens. Therefore, in upcoming generations of antibiotics, it might be employed as a nano-antibiotic.

## Introduction

Typically, physical, chemical, or biological processes are used to generate nanoparticles (NPs)^1^. Physical and chemical techniques are expensive, necessitate the use of harmful chemicals, and result in buildup and limited NPs stability. As a result, there is an urgent need to investigate NPs production procedures that are safe, eco-friendly, clean, and cost-effective. Microbial-mediated NP synthesis is a novel technique with numerous uses in agriculture, agricultural production, and medicine. Therefore, it is imperative to look for safe, cost-effective, sustainable, and clean methods of manufacturing NPs. A revolutionary method with several bio-applications is microbial-mediated NPs synthesis^2^. Nanobiotechnology is a relatively recent area of study that examines how biological processes in living organisms enable the production of nanoparticle NPs^3^. The many characteristics of NPs are what give rise to their bio-applications; safety is the most important characteristic, followed by composition, form, small size, large surface area, and charge^4–6^. The biological sources have been employed to produce NPs, including bacteria^7^, fungi^8^, actinomycetes^9^, algae^10^, plant^11,12^, and yeast^13,14^. These life forms were used as stabilizing and reducing agents to generate NPs^2^. The process of generation could take place inside the microbial cells^15^ or after their bioactive compounds, such as proteins and carbohydrates, are extracted^16^.

Endophytes are one of the many types of microbes that can produce unique bioactive compounds with significant medical significance^17^. These endophytes are microscopic microbes that live inside plants and participate in a variety of biological activities without affecting their host^18^. Endophytes are capable of producing a wide range of secondary metabolites, including amino acids, enzymes, carboxyl groups, hydroxyl groups, and other active groups with important pharmacological characteristics^19^. All of this variety in generating groups makes bio-fabrication of metal-NPs using endophytes desirable. Because these groups can be used as effective reducing/capping agents in the synthesis of green NPs^20^. Myco-fabricated NPs are produced in a less expensive, safer, and non-toxic manner by employing endophytic fungus cells rather than other endophytes. Inorganic metal-based NPs such as Ag, Au, Cu, Mn, and Zn are now employed in a variety of industries including medicines, electronics, food packaging, wastewater treatment, chemical and pharmaceutical industries, antimicrobial agents, and coating materials for medical devices^21^. Metal oxide nanoparticles (ZnO and MnO NPs) are becoming increasingly attractive and of particular interest in a wide range of biological applications, including gene transfer, biological labelling, nanomedicine, and pharmaceutical management. Because they are stable, safe, and non-toxic^21,22^. Another surprise is that ZnO and MnO NPs exhibit a variety of antimicrobial, antidiabetic, and acaricidal activities depending on their concentration, size, shape, and stability^23–25^. Many studies have previously bio-fabricated ZnO and/or MnO NPs using various microorganisms^7,26,27^. But very little studies have been done on myco-fabricating these metal oxides using endophytes. The endophytes *Trichoderma viride*^28^, *Xylaria arbuscula*^29^, and *Aspergillus niger*^30^ were previously used to fabricate ZnO NPs. Furthermore, endophytic *Trichoderma virens* was employed in the myco-fabrication of MnO NPs^7^. Remarkably, endophytes have not yet been used to generate a ZnO/MnO nanocomposite.

In an industrial bioprocessing technique, living microorganisms are used as factories to generate bioactive molecules using mathematical kinetic models. Depending on the examined microbe, a distinct kinetic model can be employed to improve the yields of the generated biomass and/or its secondary metabolites^17^. In order to optimize the medium ingredients and/or growth parameters, these models can be employed to describe, predict, develop, and evaluate the fermentation process^33^. Different fermentation systems, such as batch and fed-batch fermentation, can be used to significantly increase the biomass produced as well as its bioactive metabolites^34^. In a batch fermentation system (closed culturing system), sterile medium is inoculated with microbial cells and cultured for a specified period of time under optimal physiological conditions to achieve the highest rate of growth and largest biomass weight^35^. During the incubation period, the microbe's cells grow and go through various stages of development, resulting in a large biomass and bio-metabolites. Once all of the ingredients in the growing medium had been consumed, the batch cultivation period ended. To increase the yield of primary metabolites, the microbial exponential phase should be extended^36^. However, it should be reduced to increase the yield of secondary metabolites. In this case, the supporting ingredient(s) are continuously added in small, regular amounts for the duration of a specific period set using an alternative fermentation approach called the fed-batch fermentation mode^17^. As a result, a stage called fed-batch fermentation mode is added to batch fermentation to increase the yield that is collected^37^. Different fed-batch fermentation modes can be used to feed the medium ingredient(s) in accordance with the investigated microbe in order to maintain the nutrients at their ideal level^38^. The ingredient(s) can be added in an exponential or constant manner, using pulses or a continuous feeding mode^17^.

Microorganisms are able to grow on a massive scale quickly by adjusting the culture parameters (such as biomass levels, culture time, temperature, and pH) for synthesizing NPs with stability, size, and desired shape^39–41^. The major challenge to the commercial manufacture of bio-nanocomposites is their expensive production line. So, many studies have focused on improving the parameters of biogenic processes utilizing low-cost raw materials in order to raise the yield of bio-nanocomposites^42^. A few studies improved the properties of the biogenic reaction for some metals and metal oxide NPs^7,9,10,16^. However, until now, no one has really investigated the best fed-batch fermentation modes or any fermentation techniques for endophytic fungi, which should maximize the amounts of nanocomposites. To address these issues, we propose to build a large-scale fermentation system that will maximize the growth of isolated endophytic fungi. The efficient antimicrobial ZnO/MnO nanocomposite will then be myco-fabricated employing this fungus as a nano-factory. The specific objectives of this work are to explore the viability of employing endophytic fungal extract as a reducing/capping agent in the bio-fabrication of ZnO/MnO nanocomposite. A further significant objective will be to characterise the bio-fabricated nanocomposite using UV–Vis spectroscopy (UV–Vis), scanning electron microscopy (SEM), transmission electron microscopy (TEM), Fourier transform infrared spectroscopy (FTIR), thermogravimetric analysis (TGA), energy dispersive X-ray (EDX), and X-ray diffraction analysis (XRD). This bio-fabricated ZnO/MnO nanocomposite was then scaled up using fed-batch fermentation processes. Our work's ultimate objective is to evaluate the antimicrobial effectiveness of the bio-fabricated ZnO/MnO nanocomposite against a range of human diseases. human pathogens.

## Materials and methods

### Materials

In our work, the *Ziziphus spina-christi* leaves were collected from the grounds of Alexandria, New Borg El Arab City, Egypt. Multi-drug resistant human pathogens, including *Escherichia coli* (ATCC 10536), *Klebsiella pneumoniae* (ATCC 10031), *Pseudomonas aeruginosa* (ATCC 27853), *Staphylococcus epidermidis* (ATCC 14990), *Staphylococcus aureus* (ATCC 25923), *Streptococcus pneumoniae* (ATCC 33400), *Candida albicans* (ATCC 10231), *Candida krusei* (ATCC 6258), and *Candida tropicals* (ATCC 13803), kindly provided by the Department of Bioprocess Development (BID), Genetic Engineering and Biotechnology Research Institute (GEBRI), City of Scientific Research and Technological Applications (SRTA-city), New Borg El Arab City, Alexandria 21934, Egypt.

### Isolation of mycoendophytes

To remove dust, the collected plant's leaves were rinsed under running water for five minutes. After five minutes of surface sterilization with 3.5% sodium hypochlorite, the leaves were subjected for one minute to 70% ethanol. To eliminate any remaining sterilizer’s materials, the sample was washed once more with 70% ethanol for two minutes. It was then repeatedly rinsed in sterile distilled water for 1 min. The plant's leaves were then chopped into bits in an aseptic condition^43^. The sterile segments were placed on a potato dextrose agar (PDA) plate containing 30 mg/l of penicillin G and streptomycin sulphate^44^. To generate a control sample, water washes from the surface-sterilized section were inoculated onto PDA (antibiotic-free). These Petri plates were kept warm (28 °C) and in the dark^45^. The fungal cells started to expand after 14 days. These fungal samples were purified through the use of PDA sub-culturing techniques^19^.

### Myco-fabrication of ZnO/MnO nanocomposite and its antimicrobial properties

The isolated fungal cells were placed in a sterile Czapek-Dox broth medium (3% sucrose, 0.2% sodium nitrate, 0.01% dipotassium phosphate, 0.05% magnesium sulphate, 0.05% potassium chloride, and 0.001% ferrous sulphate) and statically cultivated for 48 h at 28 °C. The mature fungal mate was then collected using filter papers. Each fungal mat was then mixed in 50 mM PBS (pH 7.0) and subjected to 25–30 bursts of ultrasonic sonication at 40–50% duty to prepare the cytoplasm fraction. This fraction was cleaned by centrifugation for 20 min at 14,000 rpm, then stored in sterile glass screw bottles at 4 °C for further investigations^46^. The fungal extract was used as a reducing, stabilizing, and capping agents in the myco-fabricated of ZnO/MnO nanocomposite employing 1 M of each Zn (NO_3_)_2_ 0.6H_2_O and Mn (NO_3_)_2_·6H_2_O as parent compounds (precursors)^47^. To efficiently generate a ZnO/MnO nanocomposite, 50 ml of 1 M Zn (NO_3_)_2_·6H_2_O and 50 ml of 1 M Mn (NO_3_)_2_·6H_2_O were dropped all at once in 100 ml of fungal extract under shaking condition (50 rpm). The reaction's color transformed from colorless to turbid yellowish-brown when Zn and Mn ions were produced^48^. The myco-fabricated ZnO/MnO nanocomposite subsequently received an extra dose of the fungal extract (50 ml). Then the reaction's color changed from yellowish-brown to brownish-creamy. The pellet of bio-fabricated ZnO/MnO nanocomposite was then centrifuged for 25 min at 14,000 rpm, followed by three washings with distilled water. The pellet was then dried for 24 h at 70 °C in oven. The dried myco-fabricated ZnO/MnO nanocomposite was ground to a fine powder estimated at g/l and kept in capping vials^17^. The antimicrobial effect of our myco-fabricated ZnO/MnO nanocomposite was assessed using the well-diffusion method^49^.

Some multi-drug resistant human pathogens, including *Escherichia coli* (ATCC 10,536), *Klebsiella pneumoniae* (ATCC 10,031), *Pseudomonas aeruginosa* (ATCC 27,853), *Staphylococcus epidermidis* (ATCC 14,990), *Staphylococcus aureus* (ATCC 25,923), *Streptococcus pneumoniae* (ATCC 33,400), *Candida albicans* (ATCC 10,231), *Candida krusei* (ATCC 6258), and *Candida tropicals* (ATCC 13,803), were used for evaluating the ZnO/MnO nanocomposite's antimicrobial sensitivity in vitro. These pathogens were cultured for 18 h at 30 °C in sterile Muller Hinton broth medium that contained 0.2% beef extract, 0.15% starch, and 1.75% casein. Initially, Muller-Hinton agar plates were swapped with 0.1 ml of each of these culture solutions individually. Subsequently, wells with a diameter of 6 mm were drilled using a sterile well cork-borer. Then 50 µl of myco-fabricated ZnO/MnO nanocomposite was loaded into these wells. At that moment, these plates were maintained at 4 °C for a 5-h diffusion period. These plates were incubated for 48 h at 37 °C. After that, the diameter of the produced inhibitory zones (the clear zone surrounding each well) was determined using a ruler (a millimeter scale)^49^. For more studies, a tested isolate of a mycoendophytic fungus that can produce ZnO/MnO nanocomposite as a strong antimicrobial agent was selected.

### Molecular identification of mycoendophytic isolate

The selected endophytic isolate was identified by amplifying and sequencing the ITS region (ITS1-5.8S-ITS2)^50^. In brief, the recovered endophytes' pure mycelia were used to extract the complete genomic DNA utilizing the Minipreps DNA Kit from (Promega, USA). This DNA was subjected to PCR amplification of the ITS region (ITS1-5.8S-ITS2) using universal primers ITS1 (5′-TCCGTAGGTGAACCTGCGG-3′) and ITS4 (5′-CCTCCGCTTATTGATATGC-3′) using a Veriti thermal cycler (Applied Biosystems, Singapore). One unit of Taq DNA polymerase, 50 ng of template DNA, 1X PCR assay buffer, 1.5 mM MgCl_2_, 200 m of each dNTP, and 10 pmol of each primer would constitute the components of up to the 25 µl PCR reaction^51^. The temperature for the PCR was 95 °C for 5 min, followed by 40 cycles of 95 °C for 1 min of denaturation, 56 °C for one min of annealing, 72 °C for 2 min of extension, and last of all 72 °C for 10 min. According to the company's guidelines, the purified PCR product was immediately sequenced using the Model 3130 automated DNA sequencing, Genetic Analyzer, Applied Biosystems, Hitachi, Japan. The resultant sequence was compared to similar sequences using NCBI BLAST. Multiple sequence alignment was used for building a phylogenetic tree using the neighbor-joining (NJ) method throughout the MEGA10.1.7 software 2020, together with the BioEdit programme (Hall, 1999).

### Industrial biotechnological strategies to increase the yield of myco-fabricated ZnO/MnO nanocomposite

The chosen strain was grown for seven days at 28 ± 2 °C in a plate of Sabouraud dextrose (SD) agar (4% glucose, 1% peptone, and 1.5% agar). The conidia pre-inoculum concentration was achieved at 3.0 × 10^6^ conidia/ml using a 0.01% Tween 80 solution. In this investigation, a modified semi-synthetic Czapek-Dox medium was employed as the production broth medium^52^. In this medium, casein-peptone (12% N) and glucose (40% C) are used as nitrogen and carbon sources, respectively, with a N:C ratio of 10: 20. The working capacity of the used broth medium was 4.5 L, which was transferred to a 7-L BioFlo 310 bioreactor (New Brunswick Scientific, Edison, NJ, USA). The pH was initially set to 4 after sterilization process. Aeration was initially adjusted at a rate of 1 vvm during agitation speed of 160 rpm. The bioreactor was subsequently dropped with an inoculant, and it was let to run for a total of 7 days at 25 ± 3.0 °C with constant lighting. During the fermentation process, samples were obtained at regular intervals to monitor the yields of fungal cell-generated biomass and ZnO/MnO nanocomposite. The agitation speed and airflow rate were both kept above 15%. The batch-fermentation phase came to an end when the concentration of dissolved oxygen (DO) increased, which signified the depletion of the availability of a carbon source (glucose). The control sample was taken before the fed-batch phase started in order to evaluate the yields of the biomass and its myco-fabricated ZnO/MnO nanocomposite. As can be seen in Table 1, various feeding schedules were designed at this time, employing different ratios of the used nitrogen and carbon sources. The kinetics of fungal biomass and the myco-fabricated ZnO/MnO nanocomposite were described and determined using different fermentation models. The data were collected using at least three distinct experiments (n = 3) then reported as means and standard deviations (M ± SD). The statistical analysis and visualization of the controllable variables in the selected fed-batch fermentation model were performed with Origin-Lab 2021 software.

**Table 1:** Demonstrating the tested fed-batch runs using a 7-L bioreactor to maximize the yield of biomass and myco-fabricated ZnO/MnO nanocomposite with optimizing feeding strategy, feeding regime, and feeding volume.

Codes of Fed-batch runs	Feeding ingredients Nitrogen(N): Carbon (C) sources	Feeding regimes	Feeding volumes
FB1	20: 400	Constant Feeding	6.0 ml/L/hr
FB2		Exponentially Continuous Feeding	6.0–60.0 ml/L/hr
FB3		Exponentially Pulsed Feeding	6.0–30.0 ml/L/hr
FB4	1:200	Constant Feeding	6.0 ml/L/hr
FB5		Exponentially Continuous Feeding	6.0–60.0 ml/L/hr
FB6		Exponentially Pulsed Feeding	6.0–30.0 ml/L/hr

### Characterizations of myco-fabricated ZnO-MnO nanocomposite

The bio-fabricated ZnO/MnO nanocomposite's morphology was evaluated by SEM analysis (JEOL, JSM-6460LV, Tokyo, Japan). Gold was sprayed over this examined sample using a sputter coater (JOEL Ltd., Tokyo, Japan). Additionally, TEM analysis was assessed using TEM (JEOL, JSM-6460LV, Tokyo, Japan). Moreover, Shimadzu ATR FTIR-8400 S (Japan) was used to record IR spectra of ZnO/MnO nanocomposite as well as the used fungal extract. For all spectra, thirty scans were collected from 4000 to 400 cm − 1 wavelength with a 4 cm − 1 resolution. Furthermore, TGA (TG 209 F1 Libra, Germany) was used to determine thermal stability for the bio-fabricated ZnO/MnO nanocomposite under a nitrogen atmosphere (10 ml/min). Temperature scale was set from 35 °C to 700 °C with a heating rate of 20 °C/min^53^. Finally, Zeta potential of the bio-fabricated ZnO/MnO nanocomposite was analyzed by dynamic light scattering (DLS) using Horiba, SZ-100, Kyoto, Japan. The bio-fabricated ZnO/MnO nanocomposite were diluted and dispersed by the sonication process for 20 min in an ultrasonic bath and were analyzed directly using DLS instrument and the temperature was set as 25 °C during the analysis^30^. X-ray diffraction analysis of ZnO/MnO nanocomposite was carried out using Shimadzu X-Ray diffraction (7000, USA, Cu-Kα radiation). The radiation wavelength was 1.5406 Ǻ. The data were acquired in the form of 2θ versus intensity (a.u) chart.

### Evaluation of the antimicrobial capabilities of myco-fabricated ZnO-MnO nanocomposite

The antimicrobial efficacy of various doses of the scaled-up myco-fabricated ZnO/MnO nanocomposite (0, 10, 50, 90, 130, 170, 210, and 250 µg/ml) was assessed using different methods. An initial inoculate (10^6^ CFU/ml) was generated using a 0.5 McFarland turbidity standard. The pathogenic inoculates of *Staphylococcus aureus, Escherichia coli,* and *Candida albicans* were each mixed into 9 ml of sterile nutrient broth (peptone 1.5%, yeast extract 0.3%, sodium chloride 0.6%, and D-glucose 0.01%). All tubes were incubated for an 18-h period at 37 °C and 200 rpm. Subsequently, the agar-diffusion method was used to determine the inhibitory zones^54^. Depending on the macro-dilution strategy, several procedures, such as a broth-dilution test^55^ and a time-kill kinetics experiment^56^, were also utilized.

### Broth dilution assay

The National Committee for Clinical Laboratory Standards was used to determine the antimicrobial efficacy of these tested doses^57^. Briefly, 200 µl of the tested doses were mixed separately with 2 ml of the tested pathogenic inoculates. The pathogenic inoculates were cultivated in the absence of a ZnO/MnO nanocomposite generated as a control. All tubes were incubated for a 24-h period at 37 °C and 200 rpm. After that, turbidity (optical density) at 600 nm was used to check the growth of the pathogens. The biofilm inhibition percentages were then determined for each sample using the OD of the matching control as applied in Eq. (1).^58^

1 $$Biofilm \,inhibition \left(\%\right)=\left[\frac{\left({OD}_{control}-{OD}_{treated}\right)}{{OD}_{control}}\right]\times 100$$

### Time-kill analysis

The antimicrobial efficacy was evaluated by counting the number of viable cells per milliliter (log_10_ CFU/ml) using pour plates^56^. The pathogenic inoculates in 9 ml were freshly generated, followed by a full two hours of incubation at 37 °C and 200 rpm. These tubes were then loaded with 1 ml of ZnO/MnO nanocomposite at concentrations of 130 µg/ml, 170 µg/ml, and 210 µg/ml. After that, these tubes were incubated for 48 h at 37 °C and 200 rpm. The generated microbial growth was recorded over all of the cultivation periods of 0, 6, 12, 18, 24, 30, and 36 h. The sample (100 µl) was repeatedly diluted into tenfold dilutions in sterile saline (0.9% NaCl) under aseptic conditions. After that, 100 µl of the final dilution was swabbed onto nutrient agar plates, and incubated at 37 °C. Viable cells (colonies) on each plate were counted and expressed as CFU/ml after 24 h of the incubation period. As a control, nutrient agar was swabbed on a culture that was generated without ZnO/MnO nanocomposite. The time-kill curve was developed for each pathogen by plotting the log_10_ CFU/ml of surviving microbes with cultivation times (in hours)^59^. The percentage and logarithmic decreases of the pathogen’s cells exposed to ZnO/MnO nanocomposite were computed for each of the time periods using Eq. (2).^60^ The duration of the ZnO/MnO nanocomposite dosage’s bactericidal activity and its ability to completely destroy the pathogen’s biofilm were further evaluated.

2 $$\% Of \,growth\, reduction=\left[\frac{\left({{\text{log}}}_{10}{{\rm{counts}}}_{Control}-{{\text{log}}}_{10}{{\rm{counts}}}_{Treated}\right)}{{{\text{log}}}_{10}{{\rm{counts}}}_{Control}}\right]\times 100$$

### Statistics analysis

At least three different experiments were used to collect the data, which were presented as means and standard deviations (n = 3). The results of each group were statistically compared using Minitab software (® 18.1, 2017), one-way analysis of variance (ANOVA), and the Tukey multiple comparisons test (*p*-value ≤ 0.05).

## Results and discussion

### Myco-endophyte isolation for the fabrication of a ZnO/MnO nanocomposite as a potent antimicrobial agent

**Figure 1:**
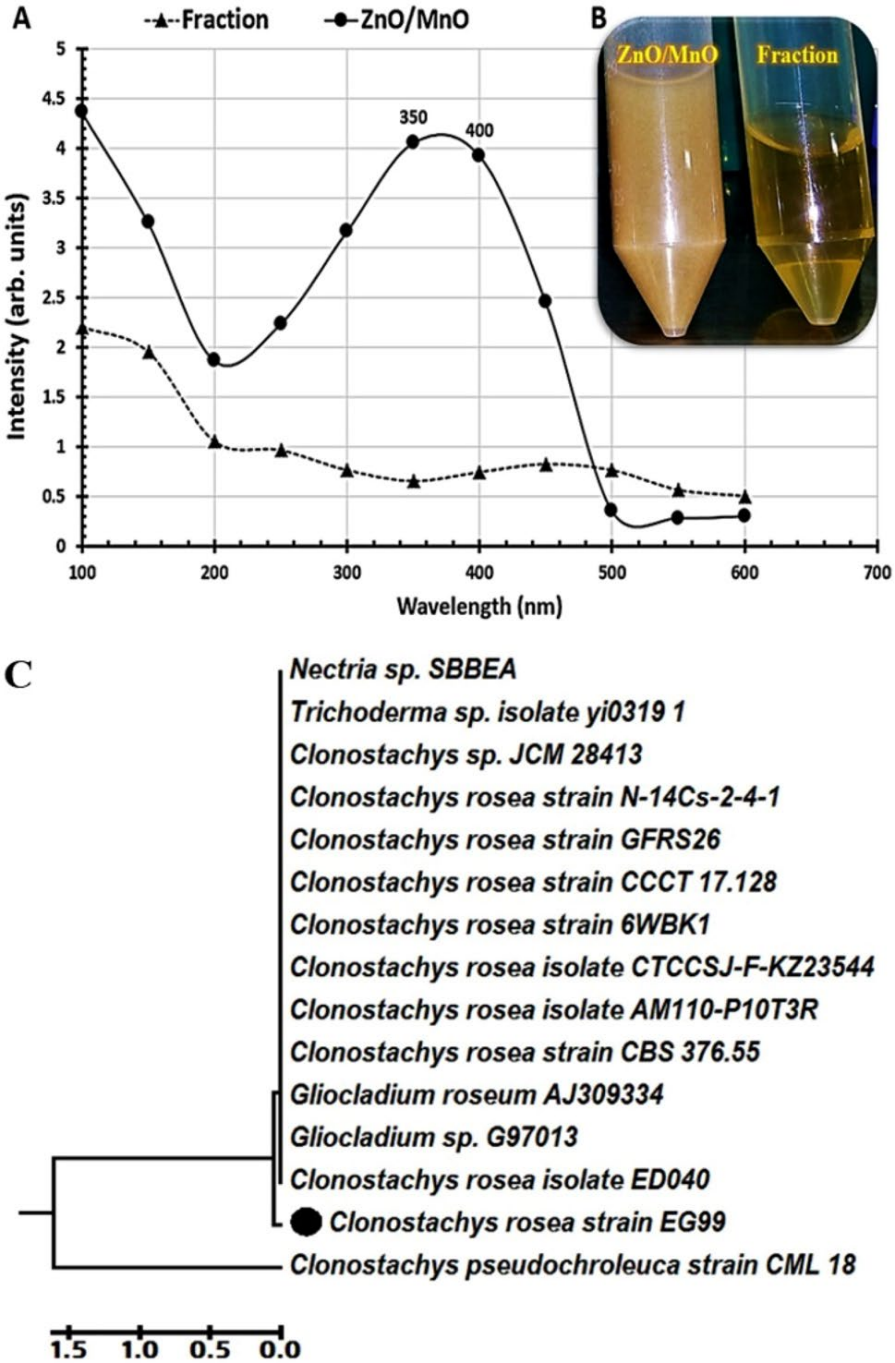
UV–Vis’s spectra of a myco-fabricated ZnO/MnO nanocomposite with surface plasmon resonance peaks at 284 and 400 nm compared to a fraction extract of the tested fungal cells (**A**), Images of the myco-fabricated ZnO/MnO nanocomposite and fungal fraction extract (**B**), the phylogenetic tree of the *Clonostachys rosea* strain EG99 (MF429774.1) in contrast to other strains (**C**). This tree was constructed using maximum probability analysis and neighbor-joining (MEGA10.1.7 software 2020). The sequence difference is indicated by a scale bar.

In our study, three endopytic fungi were isolated from *Ziziphus spina-christi* leaves. The ZnO/MnO nanocomposite myco-fabrication technique began with a titration of the fungal extract with precursors. When Zn and Mn ions were released, the reaction's color changed from colorless to turbid yellowish-brown. Subsequently, the color of the reaction changed from yellow–brown to brownish-creamy (Fig. 1B). Therefore, the produced ZnO/MnO nanocomposite was first detected by the naked eye when the response color changed as a result of the stimulation of the surface plasma vibrations^61^. The UV–Vis spectroscopy is the most widely used technique to measure the optical properties of synthesized NPs by identifying a broad absorption peak^62^. As shown in Fig. 1A, an increase in UV–Vis’s absorption intensity peaks for myco-fabricated ZnO/MnO nanocomposite with maximum absorbance at 350 and 400 nm verifies the presence of the resultant ZnO/MnO nanocomposite. The reaction's color and UV–Vis’s spectrum indicate that the goal of generating a well-reduced/stabilized ZnO/MnO nanocomposite was achieved. According to earlier studies, transitions caused the UV–Visible absorption spectra of ZnO and MnO NPs to range between 284 and 400 nm^24^. Martnez-Vargas et al*.,* also showed the fluorescence spectra of myco-fabricated ZnO/MnO nanocomposite with excitation wavelengths of 365, 369, and 371 nm^48^. Only one of the tested isolates evaluated in this experiment was able to myco-fabricate a ZnO/MnO nanocomposite. As shown in Fig. 1C, this isolate was molecularly identified as *Clonostachys rosea* strain EG99 with the accession number MF429774.1.

**Figure 2:**
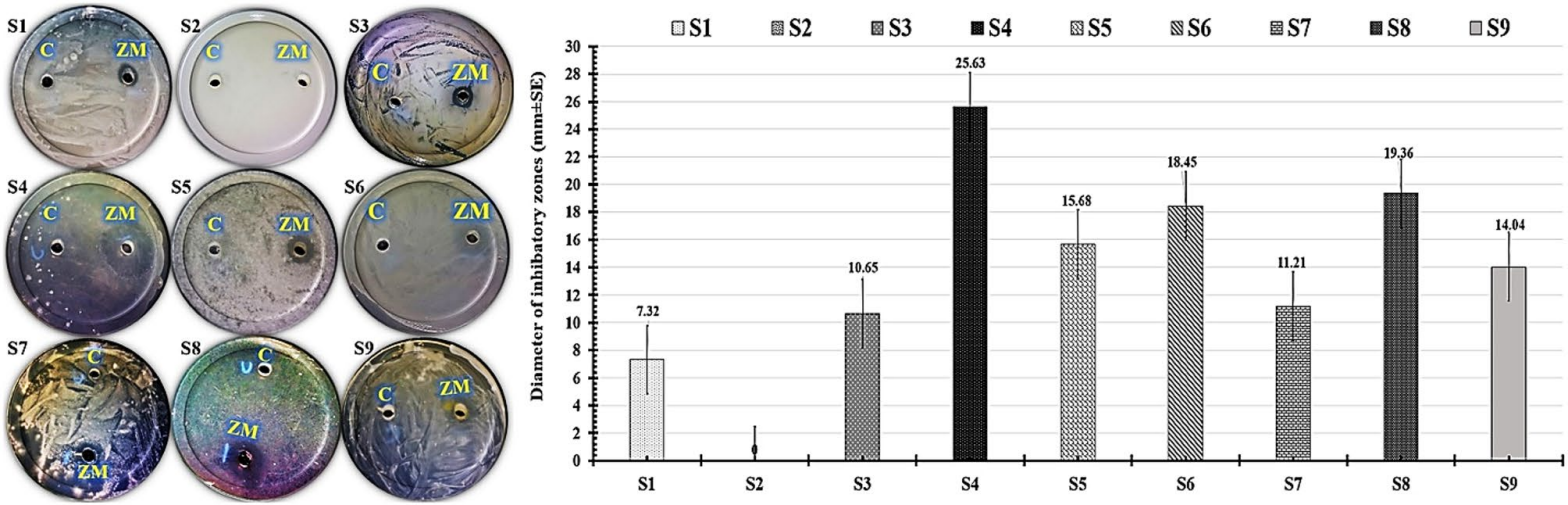
The inhibitory zones developed by the myco-fabricated ZnO/MnO nanocomposite (**ZM**) in contrast to strain EG99 extract (**C**) were shown in antimicrobial photographs and charts. The examined multi-drug resistant human pathogens, including *Candida albicans* (**S1**), *Candida krusei* (**S2**), *Candida tropicals* (**S3**), *Staphylococcus epidermidis* (**S4**), *Staphylococcus aureus* (**S5**), *Streptococcus pneumoniae* (**S6**), *Escherichia coli* (**S7**), *Klebsiella pneumoniae* (**S8**), and *Pseudomonas aeruginosa* (**S9**).

The antimicrobial efficacy of our myco-fabricated ZnO/MnO nanocomposite as well as the strain EG99 extract (control) were tested in vitro against a number of multi-drug resistant human pathogens (Fig. 2). Notably, the inhibitory zones that produced by of the myco-fabricated ZnO/MnO nanocomposite against gram-positive bacteria showed the most promising antimicrobial abilities. The largest inhibitory zones were observed in *Staphylococcus epidermidis* (25.63 mm) and *Klebsiella pneumoniae* (19.36 mm). Furthermore, the smallest inhibitory zone was observed in yeast cells like *Candida tropicals* (10.65 mm) and *Candida albicans* (7.32 mm). But the myco-fabricated ZnO/MnO nanocomposite had no effect against *Candida krusei*.

### Scaling up the produced ZnO/MnO nanocomposite

Large-scale industrial processes had a considerable impact on the efficiency of a microbial cell studied as well as its produced biomolecules. Microbes can be cultivated using two fundamental methods: liquid fermentation (a submerged fermentation system), which relies on dissolved materials, and solid-state fermentation, which utilises solid cereal-based substrates^63^. When choosing a fermentation strategy, the targeted yields and the capabilities of the examined microbial cells should be taken into consideration^64^. According to Mascarin et al., *Clonostachys rosea* strain populations can be successfully and profitably scaled up by the submerged liquid fermentation procedure^63^. So, the yields of our strain EG99 biomas with ZnO/MnO nanocomposite can be scaled up via fed-batch fermentation mode using liquid fermentation strategy (a simple and more controlled technique). The fed-batch fermentation mode consists of two stages: batch and fed-batch. Briefly, the bioreactor has been inoculated with fungal cells to initiate the batch phase. Following that, the biomass produced continued to expand rapidly until the medium substrate (glucose) was depleted completely. Increased DO levels indicated substrate consumption. Immediately after the addition of the feeding ingredients, the fed-batch phase began. In the present scenario, a variety of feeding systems have been suggested employing different ratios of the used nitrogen and carbon sources (Table 1). The kinetic behaviour of microbial biomass generation can be evaluated and specified during the fermentation mode system. The first-order equation describes the exponential growth phase, which states rate of the increased cell mass quantity at any given time^65^. The produced biomass dry weight (X_g/l), initial biomass dry weight (X_0_) at time (t) zero, maximum biomass dry weight (X_max_) and the specific growth rate (µ_hr) were used to calculate the variation in biomass dry weights per time using this formula: ∆X/∆t^32^. At log phase µ = µ_max_, and X_0_ = X_max_ were negligible, so ln X_max_ = µt as shown in Eq. (3). The computed specific growth rate during the fed-batch phase should remain constant at 0.1 to determine the feeding rate using Eq. (4).

3 $$\frac{\Delta X}{\Delta t}=\mu X; \mu =\frac{{lnX}_{max}-{lnX}_{0}}{{t}_{max}-{t}_{0}}; \to {X}_{max}={X}_{0} at t=0, \therefore ln\left[\frac{{X}_{max}}{{X}_{0}}\right]=\mu t \equiv {{X}_{max}={X}_{0}e}^{\mu t}$$

4 $$ F_{{\left( {{\text{feeding}}\; {\rm{rate}}\; {\text{ml}}/{\rm{L}}/{\text{hr}}} \right)}}  = \frac{{\mu X_{0} V_{0} e^{{\mu t}} }}{{S_{0} Y_{{{\raise0.7ex\hbox{$x$} \!\mathord{\left/ {\vphantom {x s}}\right.\kern-\nulldelimiterspace} \!\lower0.7ex\hbox{$s$}}}} }} $$

As reported in Table 2, the proficient FB4-system (constant feeding of a N:C ratio of 1: 200) demonstrated the highest biomass yield (X_max_ _89.20 ± 4.98 g/l), which in turn produced the highest yield of the myco-fabricated ZnO/MnO nanocomposite (P_max_ _79.71 ± 4.02 g/l). Subsequently, the yields of the FB5-system (Exponentially continuous feeding for a N:C ratio of 1: 200) were recorded as X_max__57.57 ± 6.47 g/l, and P_max_ _66.95 ± 0.45 g/l. Moreover, the smallest yields were observed in the FB1-system (constant feeding of a N:C ratio of 20: 400) that produced X_max__27.12 ± 2.09 g/l, and P_max__33.09 ± 1.19 g/l.

**Table 2:** Reveals the effects of different fed-batch fermentation regimens on the growth of the endophytic *Clonostachys rosea* strain EG99 that ultimately led to the fabrication of ZnO/MnO nanocomposite in comparison to the control (sample was obtained before the fed-batch phase started) via a 7 L bioreactor.

Varied specified feeding regimens' codes	Maximum dry weight (g/l ± SD)	
	Generated biomass (X_max_)	Myco-fabricated ZnO/MnO nanocomposite (P_max_)
**Control** (batch phase)	5.65 ± 1.65^d^	3.56 ± 0.97^d^
**FB 1 (**constant feeding of a N:C ratio of 20: 400)	27.12 ± 2.09^c^	33.09 ± 1.19^c^
**FB 2 (**exponentially continuous feeding of a N:C ratio of 20: 400)	33.35 ± 4.12^c^	39.75 ± 3.32^c^
**FB 3 (**exponentially pulsed feeding of a N:C ratio of 20: 400)	42.43 ± 1.46^c^	28.97 ± 1.96^c^
**FB 4 (**constant feeding of a N:C ratio of 1: 200)	89.20 ± 4.98^a^	79.71 ± 4.02^a^
**FB 5 (**exponentially continuous feeding of a N:C ratio of 1: 200)	57.57 ± 6.47^ab^	66.95 ± 0.45^ab^
**FB 6 (**exponentially pulsed feeding of a N:C ratio of 1: 200)	47.57 ± 3.43^bc^	53.89 ± 3.34^bc^

The differences in the superscript letters are statistically significant at p ≤ 0.05. R-sq (97%), adj R-sq (94.44%), and pred R-sq (88.02%).

**Figure 3:**
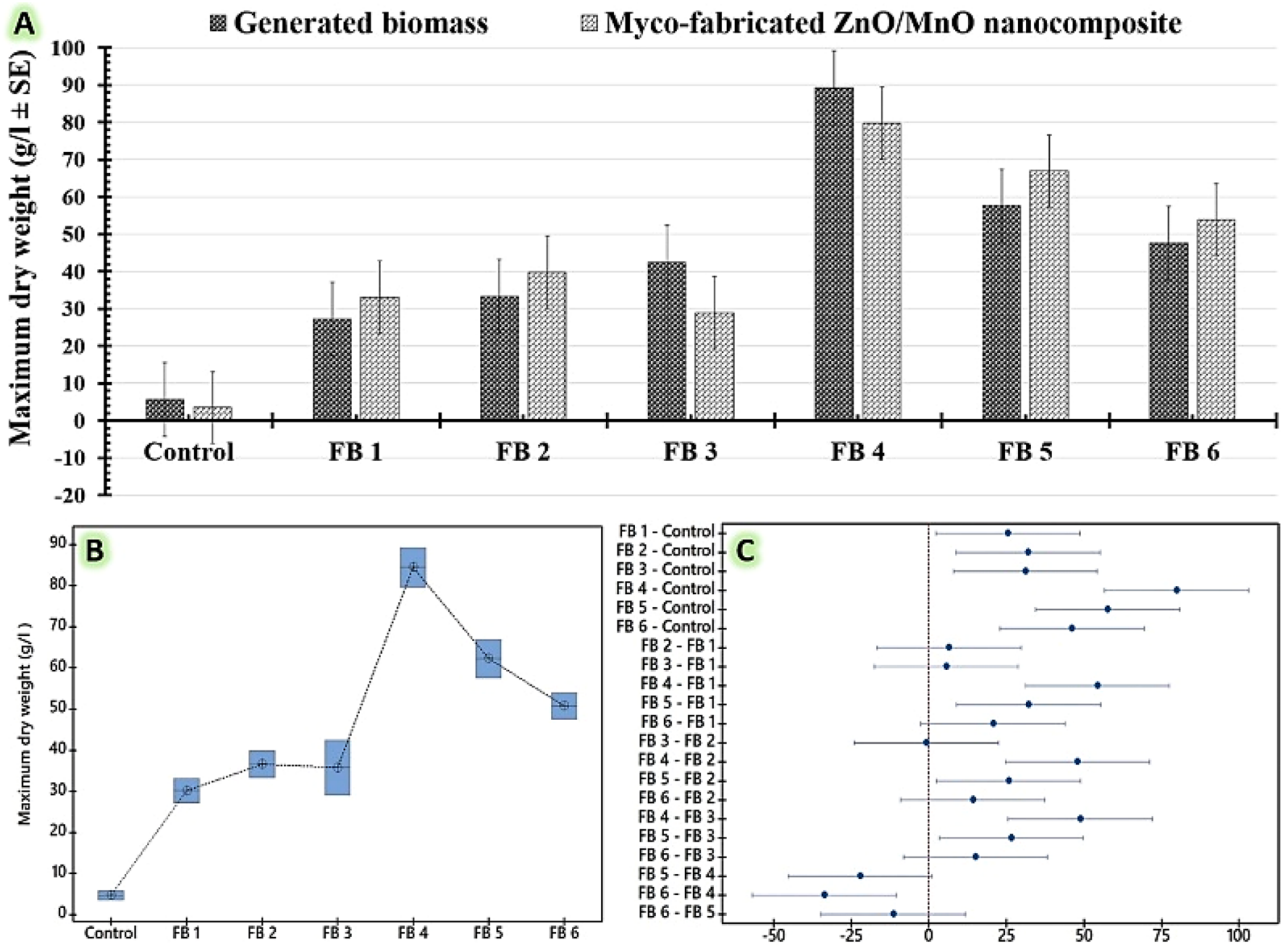
Investigate the effects of fed-batch fermentation model with varying ingredient ratios and schedules on the growth of the endophytic *Clonostachys rosea* strain EG99, and its fabricated ZnO/MnO nanocomposite in a 7 L bioreactor. The following ingredient ratios and schedules have been tested: (**FB1**) constant feeding of a N:C ratio of 20: 400, (**FB2**) exponentially continuous feeding of a N:C ratio of 20: 400, (**FB3**) exponentially pulsed feeding of a N:C ratio of 20: 400, (**FB4**) constant feeding of a N:C ratio of 1: 200, (**FB5**) exponentially continuous feeding of a N:C ratio of 1: 200, and (**FB6**) exponentially pulsed feeding of a N:C ratio of 1: 200. The control samples were collected just before the fed-batch phase started.

The constant feeding system with a N:C ratio of 1: 200 (FB4) produced the highest dry biomass weight yield, which in turn produced the highest ZnO/MnO nanocomposite output, as indicated in the chart in Fig. 3A. To identify the most effective fed-batch fermentation technique, the statistical analysis of the mean values of the computed maximum yields of the generated strain EG99 biomass and the myco-fabricated ZnO/MnO nanocomposite was calculated using the ANOVA and Tukey Post Hoc Test. The interval plot's mean values for FB1 and FB4 have been calculated to be the lowest and greatest computed yields, respectively (Fig. 3B). The adjusted confidence intervals above the 95% scale are generated using the Tukey simultaneous tests. According to our Tukey graph (Fig. 3C), the FB4 intervals don't include the zero line. As a result, this FB4 differs from the control group and the other studied fed-batch fermentation technique in statistically significant ways. The impressive outcomes revealed that the computed yields of the tested FB4 were statistically significant. The FB4-system has a distinct superscript letter compared to the other systems under examination, which makes its mean yield values statistically significant, as seen in Table 2. Additionally, the differences between FB1, FB2, and FB3 systems are not statistically significant because they share the same superscript letter.

**Figure 4:**
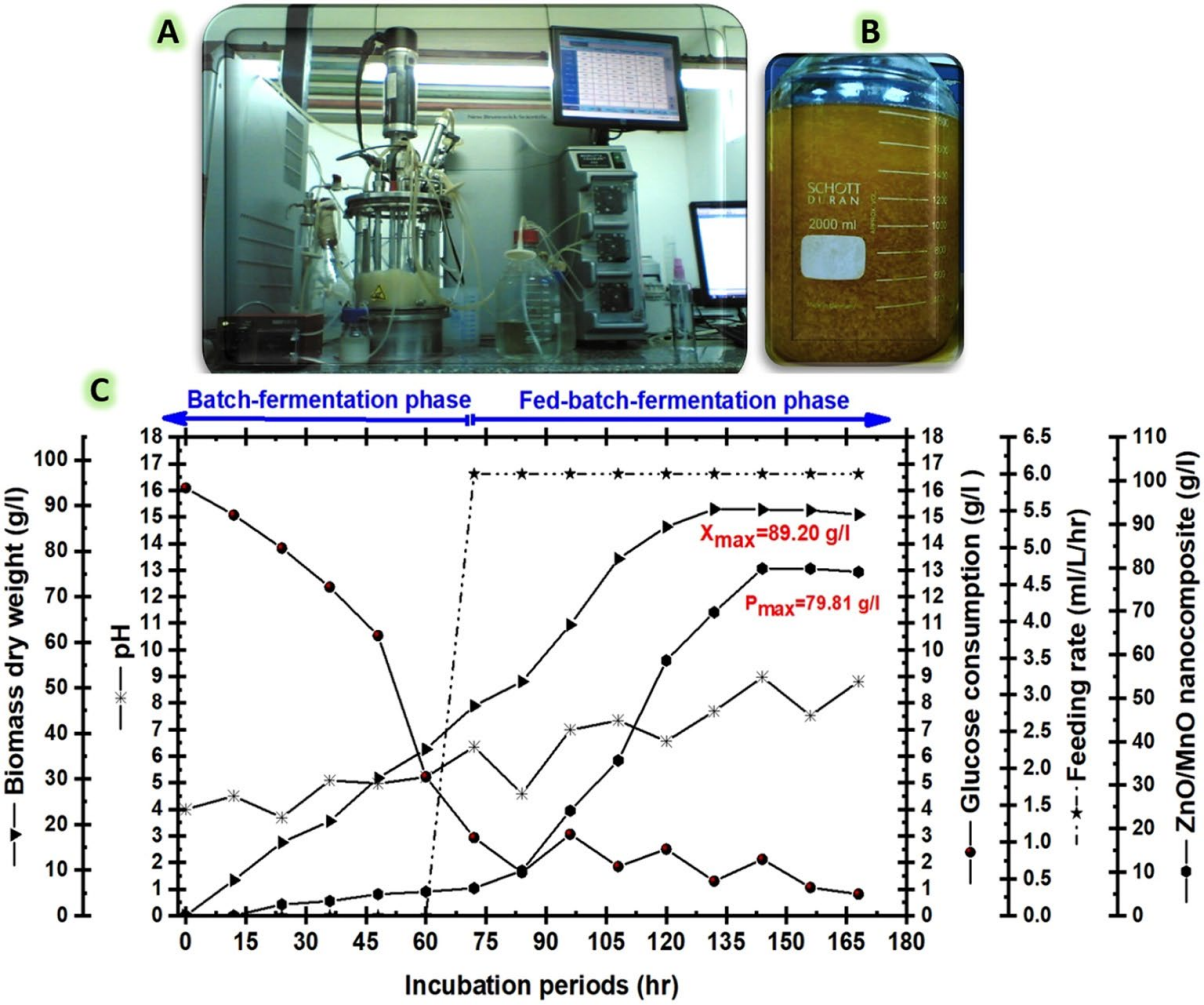
Displays (**A**) a 7-L BioFlo 310 bioreactor, (**B**) the scaled-up my-cofabricated ZnO/MnO nanocomposite yield, and (**C**) illustrates the time course for the strain EG99 growth pattern with a myco-fabricated ZnO/MnO nanocomposite yield, which describes the operation of a proficient fed-batch model with a constant feeding strategy.

The scale-up approach consists of a complex biochemical process that should be optimised in order to achieve distinct microbial yields at low production costs while at the same time improving performance^66^. Fungal cells would suffer considerable damage if the quantity of nitrogen and carbon within a bioreactor was limited because the amount of generated metabolites generally increased. These microbial cells can continue to proliferate without suffering a rupture phase if they can degrade the produced bio-metabolites and use them for energy maintenance^63,67^. In our study, a deficiency of available nutrition elements close to the end of the batch time significantly reduced the rate of biomass generation. An in-depth examination of the FB4-system, a very effective fed-batch regime, has been conducted in order to significantly prolong the biomass production period and maximise yield (Fig. 4). To control the N/C level in a 7-L stirred tank bioreactor (Fig. 4A), a constant feeding method with a N:C ratio of 1:200 was adopted. Microbial biomass productivity and the myco-fabricated ZnO/MnO nanocomposite were maximised by regulating the N/C level during the fermentation system (Fig. 4B). The maximum specific growth rate (µ_max_) was determined kinetically via Eq. (5) during the exponential growth phase using X_max_ that produced at maximum time (t_max_)^68^. At that moment, the doubling time (t_d_; hr^−1^) was calculated by Eq. (6).^69^. Correspondingly, the X_max_ (g/l ) at t_i_ and t_0_ was determined using Eq. (7)^70^ as well as, P_max_; (g/l/hr) was measured periodically according to Eq. (8)^71^.

5 $$\mu _{{max}} \left( {hr^{{ - 1}} } \right) = \frac{{\ln \left( {X_{{max}} /X_{0} } \right)}}{{\Delta t}}$$

6 $${t}_{d}\left({hr}^{-1}\right)=\frac{{\text{ln}}2}{{\mu }_{max}}$$

7 $${X}_{max}\left(g{L}^{-1}\right)=\frac{\left({X}_{i}-{X}_{0}\right)}{\left({t}_{i}-{t}_{0}\right)}$$

8 $${P}_{max}\left({gL}^{-1}{hr}^{-1}\right)=\frac{\Delta P}{\Delta t}=\frac{{P}_{max}-{P}_{0}}{{t}_{max}-{t}_{0}}$$

As shown in the Eq. (9), the growth yield coefficient (Y_x/s__ g/g) was determined using the generated X per the consumed quantities of the substrate. Furthermore, Eq. (10) was utilized to calculate the ZnO/MnO nanocomposite yield coefficient (Y_P⁄X__ g/g).

9 $${Y}_{X/S}=\frac{\Delta X}{\Delta S}=\frac{X-{X}_{0}}{{S}_{0}-S}$$

10 $${Y}_{P/X}=\frac{\Delta P}{\Delta X}=\frac{P-{P}_{0}}{{X}_{0}-X}$$

According to the data in the Table 3, the batch phase extended over 60 h with an X_max_ of 36.52 g/l, t_d_ of 0.61 h, and Y_X/S_ of 4.24 g/g. Additionally, it was reported that the P_max_ was 5.52 g/l, the Y_P/X_ was 0.51 g/g, and the µ_max_ was 0.132. Immediately after a 60-h period of cultivation, constant feeding scheme (6 ml/L/hr) with a N:C ratio of 1:200 was initiated when the glucose concentration in the late log phase reached approximately 5.23 g/l. Overall, the microbial growth kinetics released these results throughout the fed-batch phase (Table 3): the P_max_ yield was 79.81 g/l (Fig. 4C) with the Y_P/X__19.15 g/g, t_d__ 0.57 h, and the X_max_ was 89.20 g/l (Fig. 4C) with the Y_X/S__20.18 g/g. Due to the fed-batch model's accurate control of all fermentation parameters, including agitation, airflow, pH, and N/C ratio, the fed-batch yields were higher than those achieved using batch model. Finally, the fed batch's X_max_ was raised to 89.2 g/l, which was 2.44 times higher than the batch's result of 36.51 g/l. The fed-batch's P_max_ of 79.81 g/l considerably increased by 14.5 times from the batch's yield of 5.52 g/l. To the best of our knowledge, this work is the initial effort to describe the implementation of a constant feeding strategy to semi-industrially scale the manufacturing of a ZnO/MnO nanocomposite by the endophytic strain EG99.

**Table 3:** Kinetic results from the batch and fed-batch phases of the strain EG99, and these produced a ZnO/MnO nanocomposite when scaled up in a 7 L bioreactor employing a constant feeding scheme with a N:C ratio of 1:200.

Kinetic factors	Batch phase	Fed-batch phase
Operation period	60 h	96 h
pH	4.0–5.22	6.35–8.81
X_max_	36.52 g/l	89.20 g/l
µ_max_	0.132	0.01
t_d_	0.61 h	0.57 h
Y_X/S_	4.24 g/g	20.18 g/g
Y_P/X_	0.51 g/g	19.15 g/g
P_max_	5.52 g/l	79.81 g/l

In general, the feeding regime should be kinetically modulated with suitable and sufficient components in order to scale up the target yields^72,73^. The most important requirement for *Clonostachys rosea*, a saprophyte, endophyte, and mycoparasite, is a diversity of complicated carbon and nitrogen sources in order to synthesise a wide range of enzymes suited to its habitat^63,74,75^. According to some reports, monosaccharides perform better than disaccharides for cultivating *C. rosea*. Therefore, when the generated *C. rosea* was cultured in a semi-synthetic Czapek-Dox liquid medium using glucose instead of sucrose, it produced almost 50% more spores^52^. In our investigation, a well-managed bioreactor was set up for investigating the large-scale cultivation of the strain EG99. Every significant factor, including temperature, pH, agitation, dissolved oxygen, and aeration, was closely monitored during the fed-batch fermentation process. Notably, the initial medium’s pH (pH = 4.0) was increased to 8.81 after 7 days of incubation (Fig. 4). The prior reports revealed that the alkaline pH could be caused by *C. rosea*'s high biomass and spore density^52,63,74^. Additionally, our biomass of strain EG99 was scaled up massively by modifying the C:N ratio and maintaining the percentage of dissolved oxygen above 15% during the fermentation course without controlling the pH’s values. Previously, the effects of nutritional structure changes on *C. rosea* submerged culture were examined using the C:N ratio^52^. In our fed-batch model experiment, the biomass's strain EG99 and myco-fabricated ZnO/MnO nanocomposite yields were scaled up using feeding regime of a fixed N:C ratio (1:200).

### Mycofabricated ZnO/MnO nanocomposite characteristics

**Figure 5:**
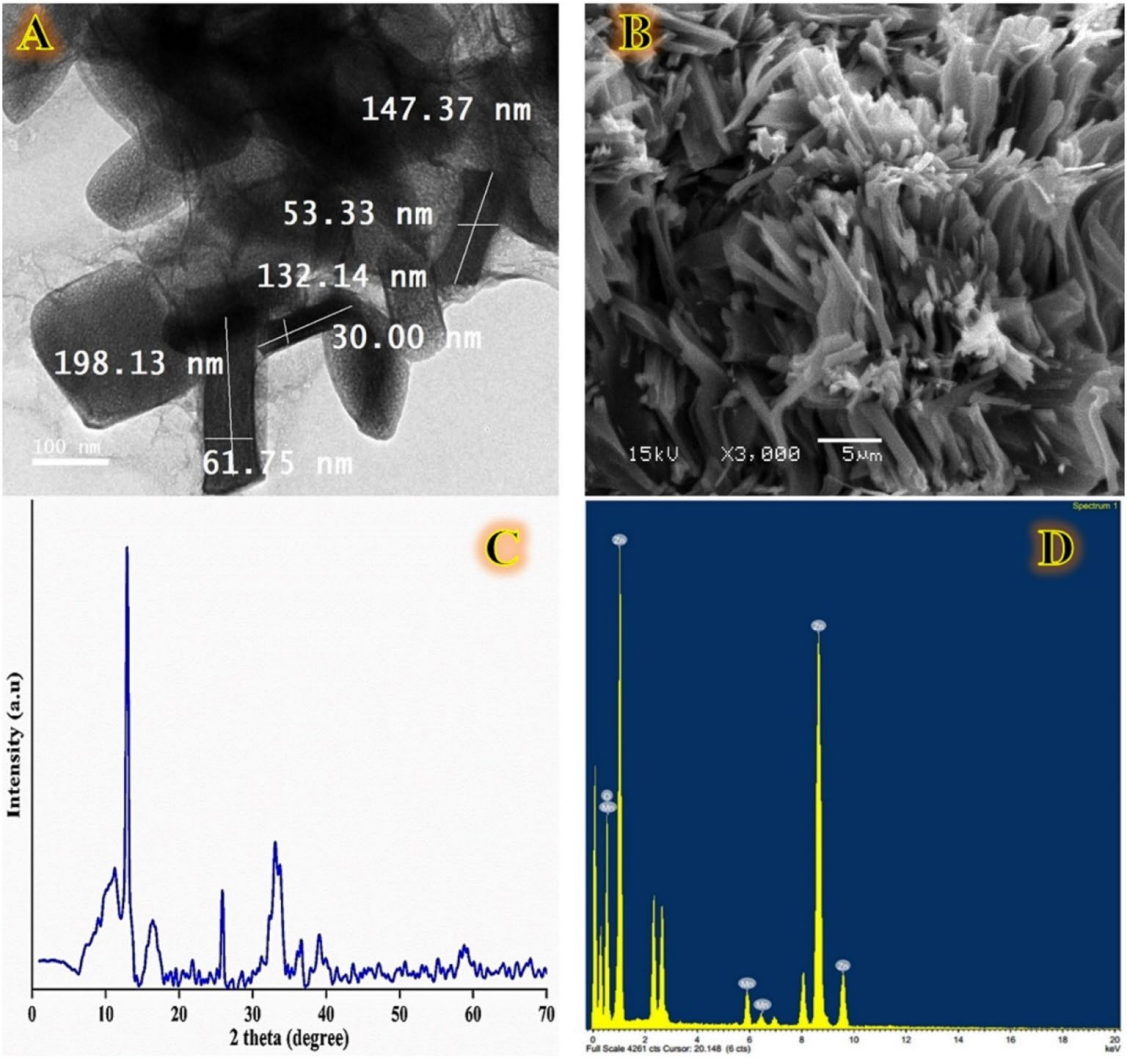
Represents the properties of a myco-fabricated ZnO-MnO nanocomposite; SEM image (**A**); TEM image (**B**); x-ray diffractive pattern (**C**), and TEM–EDX analysis (**D**).

The surface morphology of a myco-fabricated ZnO/MnO nanocomposite was well characterized by structural and morphological evaluation using SEM, TEM, EDX, and XRD analysis (Fig. 5). Our myco-fabricated ZnO/MnO nanocomposite distinguished easily due to its needle-like shape. The average length and width of the myco-fabricated ZnO/MnO nanocomposite are 132–198 nm and 30–62 nm, respectively (Fig. 5A,B). Because the size of the particles is influenced by the crystallization process, the ZnO/MnO nanocomposite that we myco-fabricated has different size distributions of particles, as seen in Fig. 5A,B. The largest crystal is made up of enormous lateral flakes, while the smallest particles show early crystal growth. Previously, the shape of ZnO@MnO_2_ nanocomposites was determined to be a mixture of cubical (ZnO) and spherical (MnO_2_) dots in clustered agglomerates^25^. Another study used a hydrothermal technique to create ZnO@MnO_2_ nanocomposites with sheet-like and nanorod morphologies of ZnO and MnO_2_ nanostructures, respectively^76^. Finally, the size and shape of the resultant nanocomposite were influenced by the capping and reducing agents utilised, the synthesis method employed, or both^83^. The chemical compositions of myco-fabricated ZnO/MnO nanocomposite were further analyzed by EDX (Fig. 5D). Elemental mappings showed that our myco-fabricated ZnO/MnO nanocomposite mainly composed of Zn, Mn and O with atomic (%) structure 60.03, 2.091 and 37.06%, respectively. The XRD analysis of the mycofabricated ZnO/MnO nanocomposite can be observed in the Fig. 5C. The XRD spectrum, which revealed a strong, intense, sharp peak at 2Ɵ = 14° and two faint peaks at 2Ɵ = 26° and 32°, confirmed the remarkable crystalline quality of the manufactured composite. Additionally, single crystals are electrolytically recovered from ingots containing more than 99 wt.% Zn, which is consistent with the EDX analysis's findings. The ZnO@MnO_2_ nanocomposites, have a mean crystallite size of 35.7 nm, which was previously determined using the Debye–Scherrer equation^25^. Our myco-fabricated ZnO/MnO nanocomposite, on the other hand, has a monoclinic crystalline structure with a crystal size of 6.22 nm.

**Figure 6:**
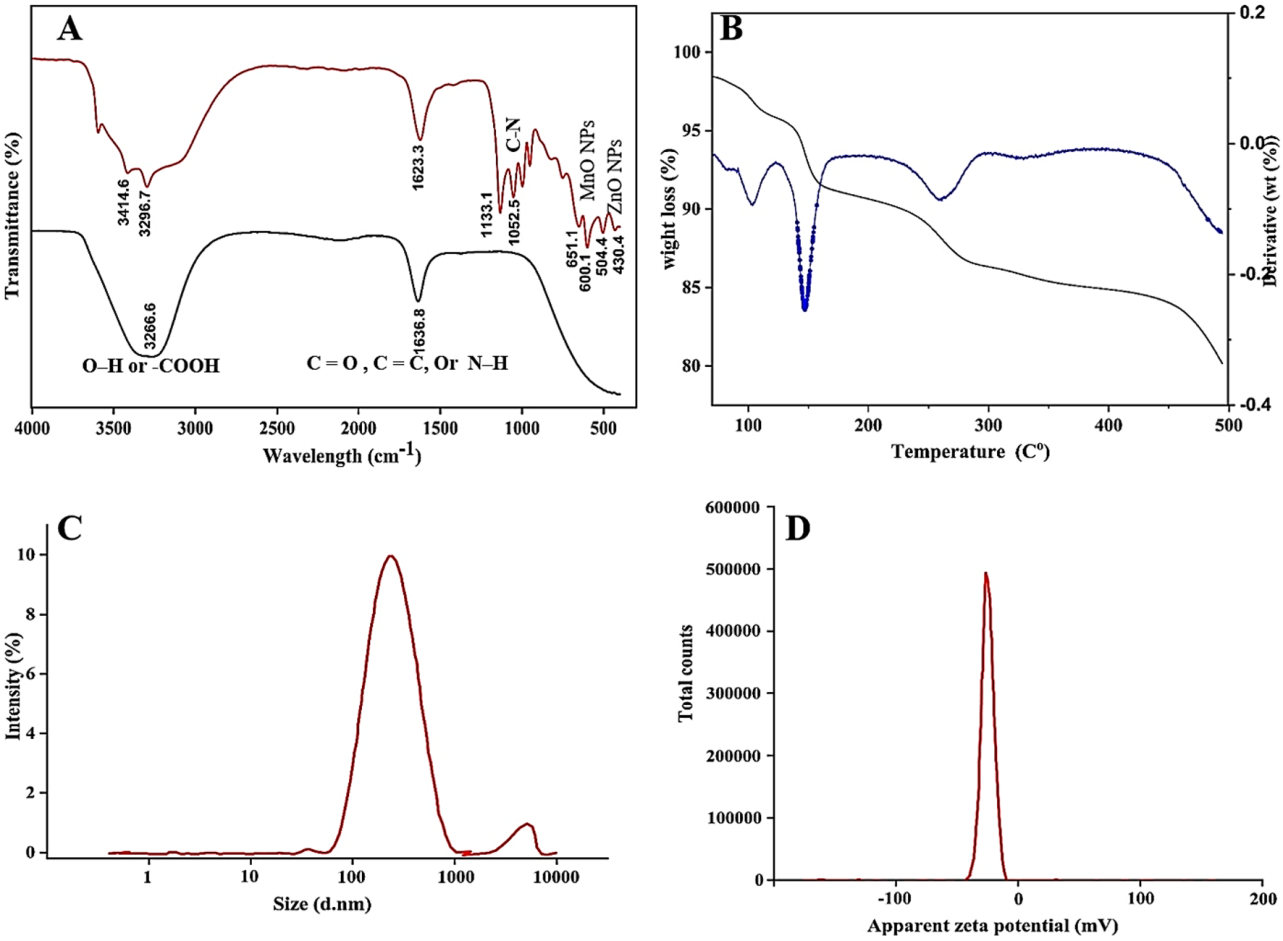
Signifies the characters of the myco-fabricated ZnO/MnO nanocomposite (**A**): FTIR spectroscopy for an endophytic extract of *Clonostachys rosea* strain EG99 (black pattern) and a mycofabricated ZnO/MnO nanocomposite (red pattern), (**B**): TGA and DTA analysis, (**C**): particle size distribution, and (**D**) zeta potential analysis.

In bio-fabrication processes, the extracted bioactive compounds generally have an effect on the size, shape, and stability of the produced NPs. In order to determine these bioactive components, the IR spectra of the produced NPs and the microbial extract were compared. FTIR spectroscopy can be utilized to identify and characterize the chemical structure of any functional groups that worked as reducing and capping agents during ZnO/MnO nanocomposite formation. In this study, FTIR spectra of the endophytic strain EG99 extract (black color) and myco-fabricated ZnO/MnO nanocomposite (red color) were shown in Fig. 6A. The absorption peak, which has been attributed to O–H stretching vibrations originating from alcohol, phenol (–OH), or acid groups (–COOH), appears at 3266.6 cm^−1^ in FTIR spectrum. The peak found at 1636.8 cm^−1^ represents amide I and amide II of the polypeptides or proteins. This absorption peak is caused by the bending vibration of the primary amine's N–H bond, the stretching vibration of C=C, or the stretching vibration of C=O in the amide 1 group. According to previous research, metal–oxygen vibration is responsible for the peaks in the 400–700 cm^−1^ area^8^. The absorption maxima of ZnO nanoparticles in our investigation are located at 525 and 440 cm^−1^. On the other hand, MnO nanoparticle absorption peaks are detected at 573 and 523 cm^−1^. Several narrow peak widths were seen in the 430–650 cm1 range, suggesting the possibility of alkene (= C–H) groups being attached to the ZnO/MnO nanocomposite that was produced. Our findings matched with previous investigations, which suggested that free amino or carboxyl groups in proteins, as well as phenol (–OH) or acid groups (–COOH), may be responsible for the nanocomposite fabrication pathway^8,13,81^. Lastly, the myco-fabrication of the ZnO/MnO nanocomposite using strain EG99 was confirmed by our FTIR data.

Figure 6B shows the thermogravimetric degradation results of the myco-fabricated ZnO-MnO nanocomposite, measured from 40 °C to 500 °C in a N_2_ atmosphere. From this figure, successive decomposition can be observed with an increase in temperature. There are mainly three regions as observed in this curve. Initial weight loss was observed at 40–120 °C due to the absorption of heat and later on successive evaporation of water vapor is seen above 100 °C. In this region, the weight loss is observed to be 4.5% of the initial weight. The second significant weight loss was noted between 120 and 180 °C as a result of the gases' thermal degradation (CO, CO2) with the elimination of functional groups, which degraded at a rate of roughly 7.2%. The last thermal breakdown of ZnO/MnO happened gradually until 443 °C. This analysis show that myco-fabricated ZnO/MnO nanocomposite is stable with an increased decomposition temperature of 400 °C. An essential physio-chemical tool for determining surface charge and colloidal stability (the repulsive forces between particles) in nano-suspensions is the zeta potential^82^. More stable NPs (less aggregation) are indicated by higher zeta potential values. On the other hand, low zeta potential levels lead to faster flocculation of NPs and reduced stability of nano-suspensions^92^. Stable nano-suspensions are defined as nanoparticles with zeta potentials greater than or equal to + 30 or −30 mV^39^. The zeta potential value is found to be (−25.2 ± 7.6 mV), indicating that the myco-fabricated ZnO/MnO nanocomposite is more stable in aqueous suspension (Fig. 6C,D). Furthermore, the average nano-rod size of our myco-fabricated ZnO/MnO nanocomposite (Z-Average (d.nm): 221.6) agrees well with the results of TEM analysis. The results obtained match up with earlier chemically determined zeta potentials of manganese-doped zinc nanoparticles^82,84^.

### In vitro antimicrobial efficacy of a myco-fabricated ZnO/MnO nanocomposite

**Figure 7:**
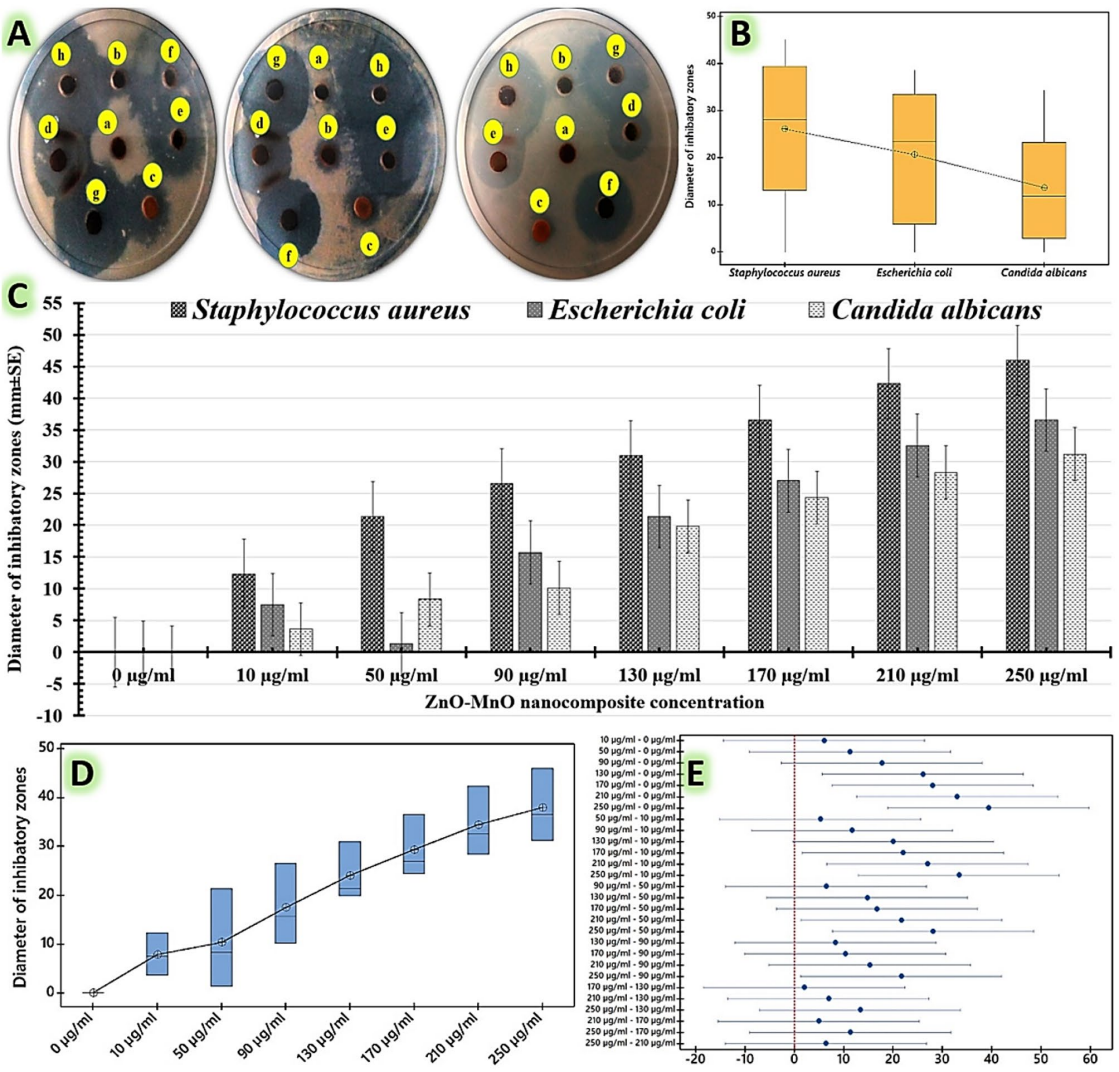
The computed inhibition zones for the antimicrobial efficiency of different doses of myco-fabricated ZnO/MnO nanocomposite against *Staphylococcus epidermidis, Escherichia coli,* and *Candida albicans* using the well-agar diffusion test, including plate images (**A**) and charts (**C**). Furthermore, the statistical analysis for the inhibitory zone diameters includes: The Box-plot graph, which compares the means for the tested pathogens (**B**) as well as the tested doses (**D**), using standard error and standard deviation boxes and whiskers via Tukey–Kramer post-hoc analysis, and (**E**) simultaneously Tukey tests for grouping mean differences. The tested doses of myco-fabricated ZnO/MnO nanocomposite include (**a**): 0 µg/ml, (**b**): 10 µg/ml, (**c**): 50 µg/ml, (**d**): 90 µg/ml, (**e**): 130 µg/ml, (**f**): 170 µg/ml, (**g**): 210 µg/ml, and (**h**): 250 µg/ml.

In these investigations, the antimicrobial activity of the scaled up myco-fabricated ZnO/MnO nanocomposite was assessed using different techniques. The doses used of myco-fabricated ZnO/MnO nanocomposite in these assessments included 0, 10, 50, 90, 130, 170, 210, and 250 µg/ml. Inhibitory halo zones were initially recognized to determine their antimicrobial qualities. The antimicrobial images (Fig. 7A) reveal that all of the tested dosages showed effective antimicrobial effects against all of the pathogens tested, in contrast to the control (0 µg/ml), where no clear inhibitory zones were observed. The ZnO/MnO nanocomposite dose of 250 µg/l generally had the highest level of antimicrobial effectiveness when compared to the other dosages (Fig. 7C). The Tukey's test means for all pairwise comparisons of the examined pathogens (Fig. 7B) as well as the tested doses (Fig. 7D) are then shown in a boxplot to find significant mean differences. The interquartile range variation is shown by each dot with whiskers in a boxplot of the investigated pathogens (Fig. 7B), which also shows inhibitory effect distributions that are statistically clustered in accordance with the recorded efficacy. Gram-positive bacteria (*Staphylococcus epidermidis*) had the highest antimicrobial efficiency, followed by gram-negative bacteria (*Escherichia coli*) and a yeast strain of *Candida albicans*. Furthermore, each dot with whiskers represents the variance in the interquartile range and exhibits inhibitory effect distributions according to the tested dosages (Fig. 7D). This box plot graph revealed that all of the data gathered on the relationship between antimicrobial activity and tested doses were statistically clustered and that the greatest efficacy was recorded at 250 µg/l of myco-fabricated ZnO/MnO nanocomposite (Fig. 7D). The Tukey simultaneous tests are used to calculate the adjusted confidence intervals above the 95% scale (Fig. 7E). The zero line is not included in the 250 µg/l of myco-fabricated ZnO/MnO nanocomposite intervals. As a result, when compared to the control group and the other examined dosages, this dose of myco-fabricated ZnO/MnO nanocomposite (250 µg/l) differs significantly in ways that are statistically significant.

As shown in Table 4, the variations between all of the tested doses of ZnO/MnO nanocomposite are statistically significant because they do not all share the same superscript letter. Additionally, the 250 µg/ml-dose has a distinct superscript letter compared to the other doses under examination, which makes its mean diameters of inhibatory zone statistically significant. The observed inhibitory zones for the 250 µg/l dose were significantly wider than those for the 170 µg/l and 210 µg/l dosages for all studied human pathogens. The moderate inhibitory zones were formed by the dosages of 90 µg/l and 130 µg/l. Furthermore, the lowest inhibitory zones were observed at the 10 µg/l and 50 µg/l dosages (Table 4). At 250 µg/ml-dose, the widest inhibitory zones were reported against *Staphylococcus aureus* (45.98 ± 6.99 mm), followed by *Escherichia coli* (36.53 ± 7.44 mm), and *Candida albicans* (31.28 ± 9.11 mm).

**Table 4:** Inhibitory zones that were formed using the well-agar diffusion method by different doses of myco-fabricated ZnO/MnO nanocomposite against *Staphylococcus epidermidis, Escherichia coli,* and *Candida albicans*.

Doses of myco-fabricated ZnO/MnO nanocomposite	Inhibition zone diameters (mm ± SD)		
	*Staphylococcus aureus*	*Escherichia coli*	*Candida albicans*
0 µg/ml	0.0 ± 0.0^d^	0.0 ± 0.0^d^	0.0 ± 0.0^d^
10 µg/ml	12.31 ± 2.14^de^	7.51 ± 0.97^de^	3.65 ± 1.21^de^
50 µg/ml	21.35 ± 2.69^cde^	1.3 ± 0.05^cde^	8.32 ± 0.97^cde^
90 µg/ml	26.56 ± 3.97^bcde^	15.69 ± 3.45^bcde^	10.12 ± 2.78^bcde^
130 µg/ml	30.98 ± 5.97^abcd^	21.34 ± 6.41^abcd^	19.78 ± 5.61^abcd^
170 µg/ml	36.54 ± 9.45^abc^	26.98 ± 8.12^abc^	24.35 ± 6.33^abc^
210 µg/ml	42.31 ± 7.88^ab^	32.54 ± 3.98^ab^	28.30 ± 9.21^ab^
250 µg/ml	45.98 ± 6.99^a^	36.53 ± 7.44^a^	31.28 ± 9.11^a^

The differences in the superscript letters are statistically significant at p ≤ 0.05. R-sq (83.55%), adj R-sq (76.35%), and pred R-sq (62.99%).

**Figure 8:**
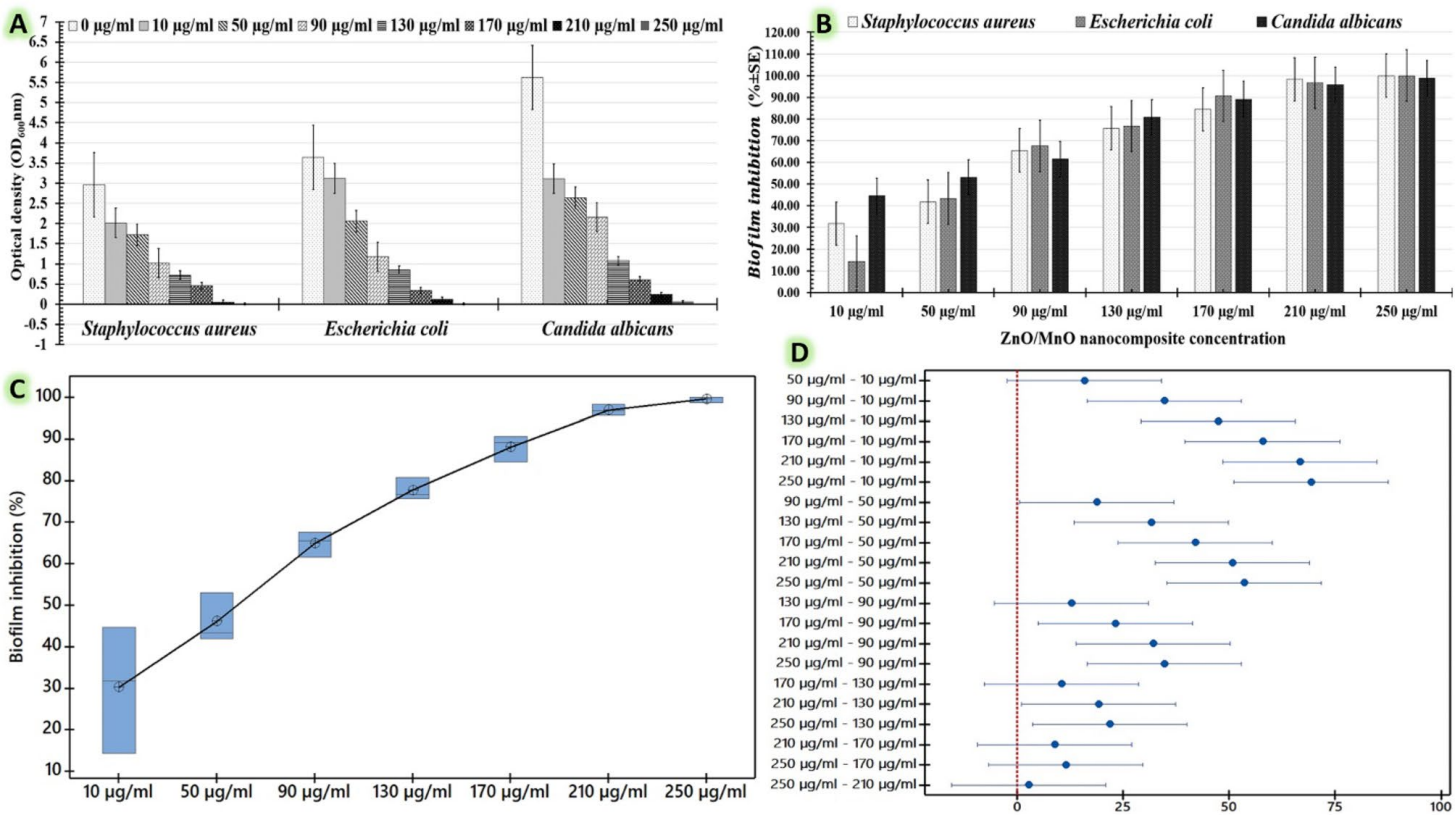
Characterizes the anti-biofilm inhibitory impact of the tested doses of myco-fabricated ZnO/MnO nanocomposite (10, 50, 90, 130, 170, 210, and 250 µg/ml) against *Staphylococcus aureus*, *Escherichia coli*, and *Candida albicans* using the microbial turbidity method. (**A**) The chart shows the recorded optical density at 600 nm of the treated pathogens with different doses; (**B**) The chart illustrates the percentage of biofilm inhibition; (**C**) The box-plot graph reflects the biofilm reduction value distributions corresponding to the tested dosages using Tukey–Kramer post-hoc analysis; and (**D**) Simultaneous Tukey tests for mean difference between doses.

Second, a biofilm development inhibition test was employed to evaluate the dose-dependent antimicrobial abilities of the investigated ZnO/MnO nanocomposite (Fig. 8). In this antimicrobial bioassay, the turbidity produced by the tested human pathogens was used to evaluate the dose-dependent anti-biofilm inhibitory effects of the tested dosages of myco-fabricated ZnO/MnO. The graphed charts (Fig. 8A,B) demonstrates that there are variations in the anti-biofilm inhibitory impacts reported against all tested human pathogens. These findings showed that the studied doses' anti-biofilm inhibitory effects were strain-dependent in addition to dose-dependent. The Tukey's test means for all pairwise comparisons are then presented in a boxplot to find significant mean differences (Fig. 8C). Each dot with whiskers represents the variation in the interquartile range in a boxplot. Anti-biofilm inhibitory effect distributions matching dosages are shown in a box plot graph using Tukey–Kramer post-hoc analysis. The higher median values were represented by the horizontal line inside the box of the resulting boxplot. Furthermore, the box plot graph verified that all of the data gathered on the relationship between anti-biofilm inhibitory impact and tested doses were statistically clustered and that the greatest efficacy was recorded at 250 µg/l (Fig. 8C). After that, a Tukey analysis was carried out with a simultaneous confidence level of 95% (Fig. 8D) to estimate the individual confidence level between each dose group. The confidence intervals for the differences between the means of the 250, 210, and 170 µg/ml groups cross the zero line, indicating that there is no statistically significant difference between the means of the three groups. The mean values of the 130 and 170 µg/ml groups are also not significantly different from one another. As indicated in Table 5, there are no significant differences between dosages of 10–50 µg/l, 130–170 µg/l, and 210–250 µg/l because these dosage pairs have the same superscript letters. The recorded mean results for 90, 130 and 210 µg/l doses are significantly different because they don't all have the same superscript letters. So, the evaluated myco-fabricated ZnO/MnO nanocomposite was most effective against *Staphylococcus aureus* (98.31 ± 0.8%), *Escherichia coli* (96.70 ± 3.29%), and *Candida albicans* (95.72 ± 0.95%) at a dosage of 210 µg/ml (Table 5). The ZnO/MnO nanocomposite's excellent antimicrobial abilities are believed to be attributed to its size, shape, and surface area, which directly interact with pathogenic cells in broth cultures^16^.

**Table 5:** Indicates the anti-biofilm inhibitory impact of the tested doses of myco-fabricated ZnO/MnO nanocomposite (0, 10, 50, 90, 130, 170, 210, and 250 µg/ml) against *Staphylococcus aureus*, *Escherichia coli*, and *Candida albicans* using the microbial turbidity method.

Concentration of ZnO/MnO nanocomposite	Multidrug-resistant human pathogens					
	*Staphylococcus aureus*		*Escherichia coli*		*Candida albicans*	
	Optical density (OD _600_ nm ± SD)	Biofilm inhibition (% ± SD)	Optical density (OD _600_ nm ± SD)	Biofilm inhibition (% ± SD)	Optical density (OD _600_ nm ± SD)	Biofilm inhibition (% ± SD)
0 µg/ml	2.96 ± 0.23		3.64 ± 0.24		5.62 ± 0.98	
10 µg/ml	2.06 ± 0.19	31.75 ± 6.75^d^	3.12 ± 0.06	14.28 ± 0.57^d^	3.11 ± 1.05	44.66 ± 1.92^d^
50 µg/ml	1.72 ± 0.56	41.89 ± 1.89^d^	2.06 ± 0.24	43.40 ± 0.65^d^	2.64 ± 1.09	53.02 ± 4.91^d^
90 µg/ml	1.02 ± 0.22	65.54 ± 0.54^c^	1.18 ± 0.35	67.58 ± 2.42^c^	2.16 ± 1.07	61.56 ± 5.83^c^
130 µg/ml	0.72 ± 0.09	75.67 ± 5.67^bc^	0.85 ± 0.33	76.64 ± 0.84^bc^	1.08 ± 0.21	80.78 ± 2.91^bc^
170 µg/ml	0.46 ± 0.03	84.45 ± 0.94^ab^	0.34 ± 0.64	90.65 ± 0.93^ab^	0.61 ± 0.02	89.14 ± 0.59^ab^
210 µg/ml	0.05 ± 0.02	98.31 ± 0.81^a^	0.12 ± 0.01	96.70 ± 3.29^a^	0.24 ± 0.14	95.72 ± 0.95^a^
250 µg/ml	0.04 ± 0.02	98.65 ± 0.85^a^	0.08 ± 0.01	97.80 ± 1.21^a^	0.17 ± 0.11	96.44 ± 0.65^a^

The differences in the superscript letters are statistically significant at p ≤ 0.05. R-sq (95.42%), adj R-sq (93.45%), and pred R-sq (89.68%).

**Figure 9:**
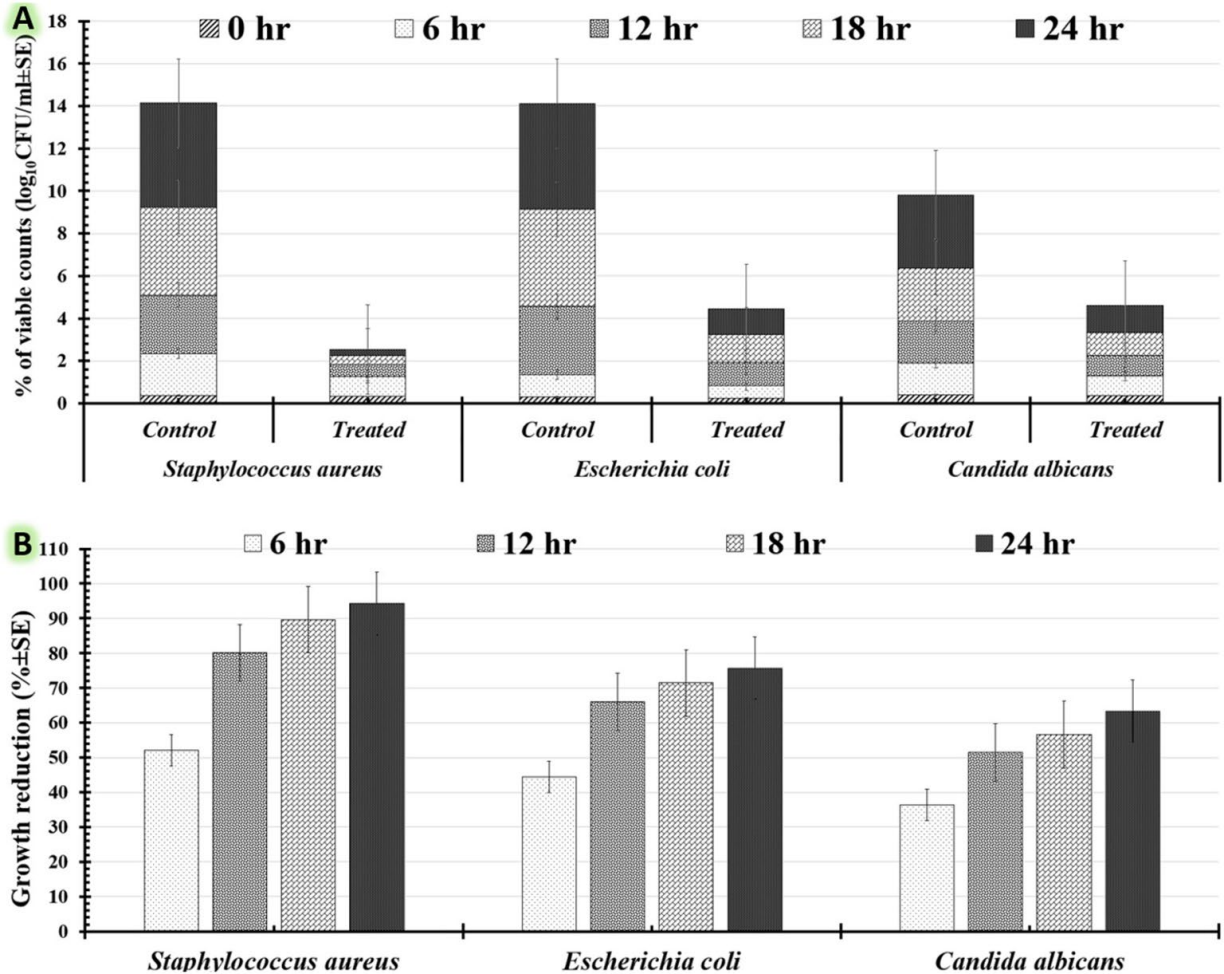
Time-kill kinetics charts comparing the growth of *Staphylococcus aureus*, *Escherichia coli*, and *Candida albicans* treated with 210 µg/ml ZnO/MnO nanocomposite to untreated controls during incubation; (**A**) viable counts (log_10_ CFU/ml ± SE), and (**B**) reductive percentage (% ± SE).

Third, the antimicrobial capacity of the myco-fabricated ZnO/MnO at 210 µg/ml was then assessed using macro-broth dilution techniques to count the number of viable cells per milliliter (log_10_ CFU/ml) of *Staphylococcus aureus*, *Escherichia coli*, and *Candida albicans*. For each time period, the percentage and logarithmic reductions of pathogen cells were calculated as shown in Table 6. The percentage of viable counts (log_10_CFU/ml) for each pathogen that developed was graphed versus cultivation times. As shown in Fig. 9A, the percentage of viable counts for the treated *Staphylococcus aureus* was lowered perfectly compared to *Escherichia coli,* and *Candida albicans* during the incubation period. So, the percentage of viable counts reduced for all treated human pathogens was recorded during the incubation period, as shown in Fig. 9B. The maximum percentage of growth reduction was reported at 96.86 ± 5.23% after 36 h of incubation for treated *Staphylococcus aureus* with 210 µg/ml ZnO/MnO nanocomposite (Table 6)*.* After 36 h of incubation, treated *Escherichia coli* and *Candida albicans* confirmed a moderate percentage of growth reduction, with their respective percentages of 81.64 ± 3.12% and 77.71 ± 3.89%, respectively. The duration of time required for the 210 g/ml ZnO/MnO nanocomposite to totally eradicate the populations of each of the tested pathogens was also determined using this time kill-kinetics experiment. The tested *Staphylococcus aureus* biofilm was destroyed after 48 h; however, the investigated *Escherichia coli* and *Candida albicans* biofilms needed 72 and 96 h to be eradicated entirely, respectively. Finally, the quantitative data gathered from each of our antimicrobial tests may serve as a starting point for figuring out the pharmacological properties of the myco-fabricated ZnO/MnO nanocomposite in vitro.

**Table 6:** Depicts an in vitro time-kill kinetics study that compares the growth of *Staphylococcus aureus*, *Escherichia coli*, and *Candida albicans* treated with 210 µg/ml ZnO/MnO nanocomposite to untreated controls and measures viable counts (log_10_CFU/ml ± SD) as well as reductive percentage (% ± SD).

Cultivation period (hr)	Pathogens treated with 210 µg/ml of ZnO/MnO nanocomposite								
	*Staphylococcus aureus*			*Escherichia coli*			*Candida albicans*		
	Viable counts (log_10_CFU/ml ± SD)		Growth reduction (% ± SD)	Viable counts (log_10_CFU/ml ± SD)		Growth reduction (% ± SD)	Viable counts (log_10_CFU/ml ± SD)		Biomass reduction (% ± SD)
	Control	Treated		Control	Treated		Control	Treated	
0	0.36 ± 0.04	0.32 ± 0.07		0.3 ± 0.23	0.25 ± 0.04		0.41 ± 0.09	0.35 ± 0.09	
6	1.98 ± 0.48	0.65 ± 0.47	52.02 ± 4.26	1.06 ± 0.78	0.59 ± 0.54	44.34 ± 5.45	1.48 ± 0.12	0.94 ± 1.24	36.32 ± 1.39
12	2.76 ± 0.98	0.95 ± 0.74	80.07 ± 3.12	3.21 ± 1.23	1.09 ± 0.04	66.04 ± 4.12	1.98 ± 0.07	0.96 ± 0.09	51.53 ± 2.98
18	4.14 ± 1.36	1.13 ± 0.95	86.62 ± 6.45	4.56 ± 1.60	1.3 ± 0.68	71.49 ± 3.12	2.51 ± 1.13	1.09 ± 1.87	56.60 ± 3.89
24	4.89 ± 1.45	1.48 ± 0.71	94.27 ± 2.59	4.98 ± 0.35	1.21 ± 0.85	75.70 ± 2.36	3.44 ± 0.78	1.26 ± 0.19	63.33 ± 2.12
30	5.35 ± 2.15	1.54 ± 0.93	95.51 ± 2.84	5.25 ± 1.24	1.19 ± 0.78	77.33 ± 2.75	4.39 ± 1.36	1.15 ± 0.57	73.793 ± 1.09
36	5.73 ± 1.97	1.28 ± 1.07	96.86 ± 5.23	5.5 ± 1.40	1.01 ± 0.74	81.64 ± 3.12	5.29 ± 0.97	1.18 ± 0.16	77.71 ± 3.89

Antimicrobial nanocomposites' effects on pathogens have not yet been completely clarified^78,85^. These antimicrobial effects of nanomaterials can be analysed, explained, and predicted using a variety of hypotheses. Due to their extremely large surface area, nanocomposites undoubtedly adhere to the microbe's cell wall and then penetrate it, damaging its permeability^78^. It's possible that these nanoparticles will bind with the phosphorus and sulphur in DNA, limiting its functionality. Additionally, the released ions bind to the thiol groups of many crucial respiratory enzymes, turning them inactive and killing pathogenic microbes^86^. These ions build up inside the microbial cells, where they may potentially have a more deadly effect by generating free radicals and oxidative stress^24^. According to published studies, the synergistic interaction between nanoparticles and the organic compounds in cell walls is the primary cause of nanocomposite increased antimicrobial efficiency^60,87,88^. Any nanoparticle's antimicrobial activity targets cell division and the respiratory chain, which ultimately results in cell death and strengthens the antimicrobial effect^88^. A microbial cell's walls and membrane serve the important function of protecting the microbe from medicinal products, such as nanomaterials. Based on the variations in how their cell walls are constructed, bacteria are categorized into different categories. The lipopolysaccharides that make up the cell envelope of Gram-negative bacteria are essentially separated into two layers^89^. In contrast, the majority of the thicker cell walls of gram-positive cells are composed of peptidoglycans, a single class of molecules^90^. When nanomaterial suspensions are loaded, it has been discovered that the generation of reactive oxygen species (ROS) increases. Some of these reactive species include hydroxyl (OH), hydrogen peroxide (H_2_O_2_), and superoxide anion (O_2_)^91^. The primary mechanism behind the antimicrobial abilities of nanoparticles is this ROS production^48^.

## Conclusion

In this investigation, endophytic *Clonostachys rosea* strain EG99 was effectively isolated from leaves of *Ziziphus spina-christi*. Its extract was used to create an antimicrobial ZnO/MnO nanocomposite by acting as a reducing or capping agent. A novel method was developed to optimize the production of myco-fabricated ZnO/MnO nanocomposite by controlling the strain EG99's growth via a large-scale fermentation system. Through a controlled fed-batch fermentation mode, the biomass production of strain EG99 and the yield of the myco-fabricated ZnO-MnO nanocomposite were scaled up. To the best of our knowledge, no other study has offered a thorough explanation of how endophytic *Clonostachys rosea* cells are produced semi-industrially using a unique feeding approach. Our results could also indicate that a novel large-scale biosynthetic method for fabricating industrial nanocomposite materials could be effectively applied on microbial services. Moreover, our findings offer strong support for the future application of the mycofabricated ZnO/MnO nanocomposite as a nano-antibiotic agent.

## Data Availability

The article includes the data that was used to support the results of a study. According to the authors, no further approvals were required to conduct research on plant material in accordance with local and institutional regulations. This study was performed in accordance with local and institutional regulations. Because it was so similar to *Clonostachys rosea*, the resultant isolate was submitted to the GenBank database (https://www.ncbi.nlm.nih.gov/nuccore/MF429774.1 ) as *Clonostachys rosea strain EG99* with the accession number MF429774.1.
